# Federated TriNet-AQ: Explainable english proficiency classification in augmented and virtual reality learning

**DOI:** 10.1371/journal.pone.0329304

**Published:** 2026-01-20

**Authors:** Chunxiao Zhang, Zhiyan Liu

**Affiliations:** 1 International Business School, Weifang Vocational College, Weifang, China; 2 College Office, Weifang Vocational College, Weifang, China; Khalifa University, UNITED ARAB EMIRATES

## Abstract

AR/VR and other immersive technologies are creating dynamic, learner-centred, and engaging language-learning environments. In these ever-changing situations, judging someone’s language abilities is difficult. Managing multimodal learner inputs, understanding model predictions, and protecting user data across distributed systems are some of the most prominent challenges. This paper proposes TriNet-AQ, a federated, interpretable deep learning architecture for classifying English competency in AR/VR platforms. This technique addresses the difficulties raised. This work employs Quantum Sinusoidal Encoding (QSE), Triaxial Attention Fusion (TAF) for multimodal feature alignment, and Quantum Modulated Integration (QMI) to enhance context-aware learning by optimizing temporal representation. Hybrid Slime Gorilla Optimisation (HSGO) aids optimization. It accelerates convergence and improves performance and economy. TriNet-AQ provides decentralized training to many clients via federated learning, enhancing privacy and flexibility. TriNet-AQ outperforms classical, fuzzy, and hybrid baselines in real-world augmented and virtual reality instructional datasets. Its accuracy is 98.5%, AUC is 0.95, and EPES is 0.89. Even when it loses 3.5% accuracy on new data, it can generalize effectively. Another SHAP-based interpretability finding is the presence of obvious feature attributions and consistent relevance across users. Statistical analysis, including Cohen’s d = 0.89 (p < 0.001), confirms the model’s significance and reliability. TriNet-AQ provides robust, easy-to-understand, and private real-time, tailored language evaluation in next-generation immersive learning environments.

## Introduction

Immersive technologies such as virtual reality (VR) and augmented reality (AR) are rapidly revolutionising online education, particularly language learning [1,2]. Augmented and virtual reality (AR/VR) technology enables students to engage with interactive, multimodal environments ideal for language acquisition. Compared to films and speeches that fail to strike an emotional chord, this is a vast improvement. Using tools such as spatial engagement, real-time feedback, and culture simulation, students participate in genuine communication experiences grounded in real-life situations [3,4]. People in immersive settings communicate with one another in a variety of methods, including writing and speech. In class, students communicate with one another via verbal responses, nonverbal cues, and facial expressions. In addition to demonstrating the students’ engagement and comprehension, using several modes of communication raises additional, well-documented assessment challenges. Conventional systems that use central databases and predetermined judgments often fall short. It tends to overlook minor patterns and may be difficult for specific individuals to understand complex material [5]. The privacy issue further adds complexity. Because AR and VR devices may capture biometric and contextual data, questions about ownership, consent, and ethical use have surfaced [6]. These concerns pertain to the use of these technologies. It becomes more challenging for students to maintain flexibility when this data is sent to central systems for processing. Furthermore, the system is more vulnerable to hacking. This highlights the significance of decentralised systems that can monitor children’s whereabouts without prying into their personal lives or requiring vital information from every device. According to [7,8], federated Learning (FL) may address the identified problems. FL allows devices and organizations train models independently and deliver encrypted changes to a common model. Unlike the conventional method of storing all data in one location. This keeps data private while allowing global collaboration. Schools assists create adaptable evaluation methods without disclosing student data. FL is difficult to integrate into immersive situations. Clear and usable learning outcomes need models that manage various data, operate with diverse learners, and are lightweight for daily devices [9,10].

There has been a recent shift toward using vocabulary lists and grammar tests as measures of language competency in AR and VR settings. Indicators of interest and comprehension in behavior are ignored. The issues with these methods make it difficult to provide students comprehensive and timely feedback [11]. An effective learning system monitors student progress and makes adjustments based on their results, such as changing the degree of difficulty of assignments or providing different types of feedback. To make the most of this adaptability, models should not limit themselves to only checking for correctness. It is able to discern trends across time, integrate data from diverse sources, and explain concepts to students and instructors. These environments need explainability for openness, trust, and educational alignment. The industry is now undergoing a shift toward decentralized, ethical, and environmentally focused learning methods to achieve these goals. These next-gen technologies prioritise transparency, adaptability, and student agency to pave the way for more fair and personalised education. As immersive technologies become increasingly common in education, assessment tools should be as responsive and human-centered as their settings. Summary of this work’s technical and domain-specific contributions to overcome these challenges:

A new unified framework, TriNet-AQ, is presented to tackle the difficulty of learning behavior modeling in immersive worlds. This design has the potential to enhance classification precision in AR/VR educational settings by leveraging quantum-modulated integration, triaxial attention fusion, and quantum sinusoidal encoding. Indicating the time, combining data from several sources, and enhancing learning in context are all possible with this approach. Following are the contributions of this work:

1. A FL system was developed to address the growing disparity in student devices and the proliferation of privacy concerns. Since each FL client is responsible for training the model independently under this decentralised method, raw data is not required. This ensures safe collaboration while also making a wide variety of user demands feasible.
2. Applying a unique hybrid approach that integrates slime mould and gorilla troop behaviour improves the optimisation process in federated systems. For dispersed, resource-constrained client systems, the metaheuristic optimiser expedites convergence and ensures consistent performance.
3. To help with educational understanding, the system includes an SHAP-based analytical layer. By identifying the most influential elements for each finding, this module provides up-to-date, comprehensive data on how students are being assessed. This facilitates a more transparent decision-making process and clarifies complex model behaviours.
4. Setting up a transparent and safe method for evaluating an individual’s real-time English competency is a crucial component of domain-specific VR/AR language instruction. The proposed method accomplishes the same objective. The ability to cater to specific needs is another advantage of immersive learning environments.

At its outset, the research examines the development of immersive language-learning technologies, namely the growing impact of VR and AR in modern classrooms. This work explains the main modelling challenges that arise in dynamic, multimodal settings. The article then describes the proposed TriNet-AQ design, including all its components and the rationale for its creation, based on these results. Afterwards, compare the framework’s performance with other baseline models and assess its accuracy and interpretability using SHAP-based explanations. Finally, the study offers practical recommendations for improving the framework’s implementation in real classrooms, based on the trial outcomes.

## Related work

The use of hybrid learning approaches enhances the dynamic and engaging nature of learning. Research in [12] classified learners’ learning styles based on their actions using artificial intelligence. It can meet each student’s needs by creating individualised lesson plans. According to their findings, [13], web mining techniques made it easier to evaluate behaviour. Because of their centralised, static architectures, both models struggle to operate in AR/VR environments, where learning co-occurs across separate domains. To enable the use of decision trees to categorise learners, the authors enhanced the Felder-Silverman Learning Style Model (FSLSM), as mentioned in [14]. Applications in virtual and augmented reality that require real-time, multimodal input demonstrated that the method was inadequate. The reason for this was that the technique relied on a limited set of samples and data types. In [15,16], the hybrid approach combining fuzzy logic and neural networks had difficulties with large FSLSMs and high-dimensional inputs, such as gaze direction or the dynamic nature of movements. Fuzzy classification trees and Bayesian networks both agree that text-based online discussion groups and chat rooms are beneficial. Despite using verbal data rather than sensor-based signals, it employed immersive learning approaches and engaged a large number of pupils. While a fuzzy logic system has been developed to simplify model comprehension, its use in AR/VR has not yet been tested [17,18]. Despite improving prediction granularity using dialog-based information, the fuzzy tree technique in [19] only worked for unimodal situations. A more formal technique to organize behaviors, fuzzy C-means, matched behavioral data with FSLSM categories. However, its static and unsupervised nature made it tougher to adapt to learners’ shifting behaviors throughout immersion activities.

The adaptive e-learning systems mentioned in [19,20] use AI and knowledge-level modeling to personalize the contents given to each learner. The adaptive feedback in these methods came from quizzes and surveys, rather than VR or AR-enhanced instruction. Furthermore, it failed to react instantly to data collected by sensors. Automatically classifying learning styles in traditional LMSs was our goal using decision tree and Bayesian algorithms, similar to what was done in [21]. Even with perfect accuracy, these models are unable to provide federated learning or cross-device sharing techniques that protect user privacy. In order to predict learning style from visual data, the CNN-based model [22] assisted with feature extraction. In the field of visual analytics, this research was carried out. Symbolic thinking and explainability, which are necessary for better transparency in instruction, were missing from the method, even if it was sound. While the deep multi-target architecture mentioned in [23] improved prediction accuracy, its inability to provide understandable feedback rendered it ineffective for instructor-led teaching. Although [24] demonstrated the significance of emotional signals in LMS logs, it was unable to fulfill the interaction requirements of AR and VR environments.

A hybrid ensemble model that incorporates several classifiers for performance forecasting—including support vector machines (SVM), multi-layer perceptrons (MLP), and others—was shown to successfully anticipate student outcomes, as mentioned in [25] This system was not intended to comprehend dynamic behavior sequences. A hybrid architecture [26] addresses temporal aspects by modeling sequential patterns using attention-based RNNs and SVM. However, university ID card swipes did not accurately represent the complex, embodied interactions of immersive language learning. Game-based learning methods also motivated and engaged students. Research in [27] found that Quizlet-based gamified learning for TOEIC vocabulary acquisition increased student attitudes but lacked multimodal flexibility and real-time feedback. The [28] fuzzy logic recommender framework offered tailored learning support in programming, but was not adaptable to immersive language-based settings.

The potential of mobile platforms in promoting learner autonomy was demonstrated by the mobile VR-integrated learning system MGVR-ELS [29], which was evaluated using conventional pre- and post-testing, as well as ANOVA. It lacked explainability and federated learning aids. A multimodal fuzzy system in [30] successfully classified data using ANFIS and SWOT analysis, but it was limited to structured input data and lacked adaptation to real-world learner behaviors. In [31], a hybrid model incorporating Bi-LSTM, fuzzy AHP, and evolutionary algorithms was presented to dynamically adjust game complexity. While this solution offered learner-specific customisation, it failed to solve federated training and device-agnostic scalability, which are crucial to AR/VR-based educational systems.

While existing approaches as shown in Table 1 highlight the promise of explainable, federated frameworks for immersive learning, our technique achieves results equivalent to TriNet-AQ by integrating triaxial attention with quantum-inspired encoding within a coherent federated framework.

**Table 1:** Summary of related work in hybrid learning for AR/VR-based language education.

Method	Objective	Limitations	Ref.
AI-based classification	Detect learning styles using behavioral features	Static, centralized infrastructure; lacks adaptation to AR/VR	[12]
Web mining + AI	Enhance behavioral data extraction for learning personalization	Not designed for immersive or real-time systems	[13]
Decision Trees (FSLSM)	Classify learning styles using Moodle logs	Small dataset, unimodal, not multimodal or adaptive	[14]
Fuzzy Logic + Neural Networks	Hybrid classification across FSLSM dimensions	Limited feature scope; lacks scalability for high-dimensional data	[15]
Bayesian Networks	Model educational behavior from forum/dialogue text	Limited to verbal/text input; lacks sensor integration	[16]
Fuzzy Classification Trees	Predict learning behaviors from natural dialogue	No multimodal support; unimodal limitation	[17]
Fuzzy Logic Framework	Improve interpretability of learner classification	Not validated in immersive/spatial contexts	[18]
Fuzzy C-Means Clustering	Cluster behavioral traits aligned to FSLSM categories	Static and unsupervised; not real-time responsive	[19]
AI + Knowledge Modeling	Adaptive feedback via learner modeling	No real-time sensor integration; lacks AR/VR adaptability	[20]
Decision Tree + Bayesian Inference	Automated style detection in LMS	No federated learning; not privacy-preserving	[21]
CNN-based feature extraction	Predict learning styles using visual analytics	No symbolic reasoning or explainability	[22]
Deep Multi-Target Networks	Improve prediction precision in adaptive learning	No interpretability; lacks educational transparency	[23]
Affective Modeling via LMS logs	Detect emotional states from learning behavior	Not suitable for interactive or sensor-rich platforms	[24]
WVC-SVM-MLP (Ensemble)	Forecast student performance using hybrid classifiers	Ignores sequential/dynamic learning behavior	[25]
Attention-based RNN + SVM	Sequence modeling of learning timelines	Non-embodied data (e.g., card swipes), lacks interactivity	[26]
Quizlet-based Gamification	Increase learner motivation for vocabulary learning	No multimodal or adaptive support	[27]
Fuzzy Logic Recommender	Personalized guidance for programming education	Domain-specific; not language/immersive adaptable	[28]
VR-integrated MGVR-ELS	Evaluate learner autonomy in mobile VR	No explainability or federated learning support	[29]
ANFIS + SWOT (Fuzzy Logic)	Multimodal proficiency classification	Relies on structured inputs; lacks real-time adaptation	[30]
Bi-LSTM + Fuzzy AHP + GA	Adaptive difficulty tuning in gamified learning	No federated design; lacks cross-device scalability	[31]

## Proposed system model

**Fig 1:**
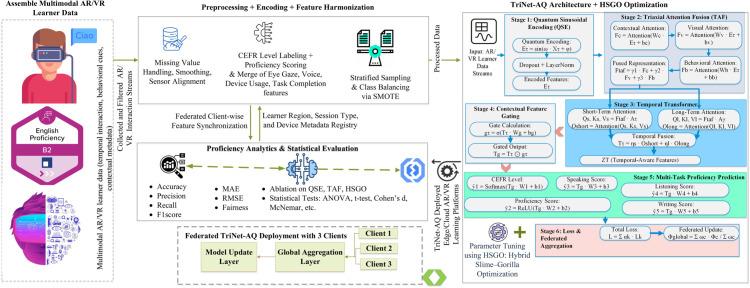
Proposed framework for English language proficiency classification in AR/VR-based curricula.

The paper introduces TriNet-AQ, a deep learning architecture designed to assess students’ English language skills within a VR- or AR-based course of study. This approach considers privacy concerns, model knowledge, data from various learning methods, and dynamic interactions. Combining visual, behavioral, and contextual data, Triaxial Attention Fusion (TAF) and Quantum Modulated Integration (QMI) enhance feature representation based on situational significance. Both Quantum QMI and QSE aim to enhance feature representation in general and capture temporal dynamics, respectively. This work aims to use the hybrid metaheuristic Slime-Gorilla Optimization (HSGO) to connect devices across different areas. With federated learning, clients can be trained without transmitting raw inputs, thereby safeguarding sensitive data. The use of a SHAP-based interpretability module to provide students with constructive criticism aids self-awareness and objective-setting training. The suggested framework is well-organised and has modules, as seen in Fig 1. Moreover, it provides a high-level summary of each area, formal mathematical equations, and an account of the methods’ applications. This method provides a better idea of how TriNet-AQ can offer privacy-protecting, easily understandable language tests.

### Data collection and preprocessing

The dataset used in this study was sourced from a publicly available Kaggle repository and generously provided by the Royal Brisbane and Women’s Hospital (RBWH) in Queensland, Australia, in collaboration with the Australian Digital Health Agency [32]. Session-level interaction records from a significant English language learning program using augmented and virtual reality platforms are anonymised. Using immersive instructional technology, a network of schools and institutions collected data over a 14-month period. Each student’s activity in the AR/VR module is discussed, and it records a wide range of actions about language competency and use. The dataset captures the intrinsic diversity across learners and devices and contains data from real deployment settings, making it suitable for federated learning. The data was processed in compliance with privacy rules, and the Queensland Health Ethics Committee approved its use and sharing. The creation of privacy-aware models in digital education is initiated using a dataset of 252,782 elements, as shown in Table 2.

**Table 2:** Overview of dataset features and their distribution characteristics.

Feature Category	Feature Names	Distribution Description
**Learner Metadata**	user_id, age_group, gender, native_language, education_level, country_region	Categorical features with unbalanced distributions; the majority of users fall within the 18–25 age group and ’Undergraduate’ education level.
**Device and Context**	device_type, internet_stability_score	Device types are skewed toward VR headsets, with internet stability varying, resulting in a higher frequency of mid- to low-range scores.
**Session Interaction Metrics**	session_duration, number_of_sessions, avg_interaction_depth, environment_type, gesture_count, response_latency, task_completion_rate, replay_count, user_feedback_score	Mixed distributions; session durations follow a clipped normal pattern, while gesture counts and replay frequency show right skewness.
**Sensor-Derived AR/VR Metrics**	eye_gaze_duration, head_movement_variance, attention_span_score, navigation_accuracy, voice_command_usage	Derived from immersive devices, features display varied ranges with non-uniform behavioral patterns across participants.
**Language Skill Scores**	reading_accuracy, listening_comprehension_score, speaking_fluency_score, pronunciation_score, vocabulary_usage_score, writing_score, grammar_accuracy, translation_skill_score	Continuous scores with heterogeneous distributions; proficiency varies widely with visible performance clustering in intermediate bands.
**Target Labels**	CEFR_level, proficiency_score, classified_proficiency, task_prediction_speaking, task_prediction_listening, task_prediction_writing	Multi-task labels encompass both categorical and continuous targets, with class imbalance evident, particularly across CEFR proficiency levels.

### Preprocessing and feature representation using the NAAL framework

The preprocessing pipeline we provide is called NAAL, an acronym for “Normalisation then Aggregation then Adaptation Layer" [33]. The objective of this pipeline is to facilitate the development of scalable, ethical, and privacy-conscious distributed AR/VR education systems that model learner behaviour. The four most prevalent issues in federated educational contexts are the primary focus of this pipeline. There are several issues, including uneven data distribution across nodes, learners following a specific sequence, feature spaces that are too large, and proficiency labels that are not evenly distributed across classes. The four components that comprise NAAL are time-based biometric aggregation (FedNorm), adaptive resampling (ARBR), context-aware feature selection (CIWS), and federated normalization. All of these components work hand in hand. To ensure the model is as suitable, generalizable, and easy to understand across as many learning environments as possible, each step in preparing the learner data is critical. The many components of the NAAL Framework are described in Algorithm 1.

**Algorithm 1 NAAL: Normalization–aggregation–adaptation layer for federated AR/VR educational data.**

**Require:** Raw learner dataset $𝒟=\{{𝐱}_{i},{𝐲}_{i}{\}}_{i=1}^{N}$ from *K* federated clients

**Ensure:** Preprocessed feature tensor $𝐗\in {\mathbb{R}}^{N\times p^{\prime }}$, label tensor $𝐘\in {\mathbb{R}}^{N\times 5}$

1: **for** each client *k* = 1 to *K* **in parallel do**

2:   **Step 1: Federated One-Hot Encoding**

3:   **for** each categorical attribute *c* in client *k* **do**

4:    Transform *c* into one-hot vector ${𝐳}_{u}$

5:   **end for**

6:   **Step 2: FedNorm (Federated Normalization)**

7:   **for** each numerical feature *x*_*r*_ in client *k* **do**

8:    Compute local mean $\mu_{r}^{\left(k\right)}$ and std $\sigma_{r}^{\left(k\right)}$

9:   **end for**

10:   Securely aggregate global mean $\mu_{r}^{\text{(global)}}$ and std $\sigma_{r}^{\text{(global)}}$

11:   **for** each *x*_*r*_ in client *k* **do**

12:    Normalize: $x_{r}^{\prime }\leftarrow \frac{x_{r}-\mu_{r}^{\text{(global)}}}{\sigma_{r}^{\text{(global)}}}$

13:   **end for**

14:   **Step 3: Temporal-Biometric Aggregation**

15:   Construct session matrix ${𝐒}_{u}\in {\mathbb{R}}^{h\times p}$

16:   Compute temporal mean ${𝐀}_{u}=\frac{1}{h}\sum_{j=1}^{h}{𝐒}_{u,j}$

17:   Compute session variance ${𝐁}_{u}=\sqrt{\frac{1}{h}\sum_{j=1}^{h}({𝐒}_{u,j}-{𝐀}_{u}{)}^{2}}$

18:   Concatenate ${𝐀}_{u}$ and ${𝐁}_{u}$ to learner profile

19:   **Step 4: Contextual Feature Selection (CIWS)**

20:   **for** each feature $\chi_{t}$ **do**

21:    Estimate mutual info $\Lambda_{t}$ using local joint probabilities ${\mathbb{Q}}_{t,y}(u,v)$ and context score $\xi_{b}$

22:    **if** $\Lambda_{t}\geq \tau$ **then**

23:     Mark $\chi_{t}$ as retained

24:    **end if**

25:   **end for**

26:   **Step 5: Adaptive Resampling (ARBR)**

27:   **for** each sample *i* in client *k* **do**

28:    Compute label rarity $\pi_{z_{i}}$ and informativeness $\kappa_{i}$

29:    Assign weight $\omega_{i}=\frac{1}{\pi_{z_{i}}}\cdot \log(1+\kappa_{i})$

30:   **end for**

31: **end for**

32: **Step 6: Construct Final Tensors**

33: Form **X** by combining selected, aggregated, and normalized features across all clients

34: Form multi-label outputs $𝐘=\{y^{\left(1\right)},y^{\left(2\right)},y^{\left(3\right)},y^{\left(4\right)},y^{\left(5\right)}\}$

35: **return** 𝐗,𝐘

#### Federated encoding and normalization (FedNorm).

Preprocessing begins by numerically encoding categorical data, such as the learner’s location, device type, or level of education. This becomes feasible with one-hot encoding. With this update, we can be confident that no hierarchical sorting or unexpected category treatment will occur. Elements of the encoded vector ${𝐳}_{u}\in {\mathbb{R}}^{g}$, where *g* represents the count of different categories, are defined element-wise when a learner *u* is considered:

$$z_{u,j}=\left\{ \begin{array}{cc} 1, & \text{if learner }u\text{ is associated with category }j\\ 0, & \text{otherwise} \end{array}\right.\;\text{for }j=1,2,\ldots ,g$$ (1)

This ensures that the schema is shown in all federated network institutions. Task duration, interaction speed, and evaluation results are all continuous variables that are normalized using the FedNorm method. Decentralized normalization is accomplished using this method. By safely collecting local client data to determine scaling parameters, FedNorm safeguards users’ privacy. The following is used to standardise a numerical attribute known as *x*_*r*_:

$$x_{r}^{\prime }=\frac{x_{r}-\mu_{r}^{\text{(global)}}}{\sigma_{r}^{\text{(global)}}}$$ (2)

The feature *x*_*r*_’s federated mean and standard deviation is obtained by using client-side summaries rather than raw data. They are identified as $\mu_{r}^{\text{(global)}}$ and $\sigma_{r}^{\text{(global)}}$ respectively. This guarantees both continuous expansion and the preservation of data sovereignty at each site.

#### Temporal-biometric aggregation for behavior modeling.

Over the course of many sessions, students’ actions while using AR and VR can vary. To capture the dynamics that change over time, NAAL can use a matrix ${𝐒}_{u}\in {\mathbb{R}}^{h\times p}$, where *h* is the total number of sessions and *p* is a physiological or behavioral characteristic like head motions, gesture frequency, or gaze focus. Two possible statistical overviews derived from this matrix are:

$${𝐀}_{u}=\frac{1}{h}\sum_{j=1}^{h}{𝐒}_{u,j},\;{𝐁}_{u}=\sqrt{\frac{1}{h}\sum_{j=1}^{h}({𝐒}_{u,j}-{𝐀}_{u}{)}^{2}}$$ (3)

A vector ${𝐀}_{u}$ is used to monitor changes in people’s behaviour over time. In contrast, the differences between the sessions are represented by the vector ${𝐁}_{u}$. Discovering shifts in participation could be possible with this. These statistics, when combined with static data, aid in building a profile of the actions and mental processes associated with each individual learner.

#### Feature selection based on contextual importance (CIWS).

It is crucial to select only the most relevant and applicable qualities from the vast amount of data obtained. Contextual Importance-Based Weighted Selection is the selection protocol used by the National Association of American Leagues (NAAL). The weighted relevance score $\Lambda_{t}$ is generated by using the mutual information for each candidate feature $\chi_{t}$ and prediction target *y*.

$$\Lambda_{t}=\sum_{u,v}{\mathbb{Q}}_{t,y}(u,v)\cdot \log\left(\frac{{\mathbb{Q}}_{t,y}(u,v)}{{\mathbb{Q}}_{t}(u)\cdot {\mathbb{Q}}_{y}(v)}\right)\cdot \xi_{b}$$ (4)

In this equation, the quantity ${\mathbb{Q}}_{t,y}(u,v)$ represents the collective likelihood of the feature value *u* and the label result *v*. The behavioural consistency score, generated via unsupervised clustering within each client, is likewise represented by the symbol $\xi_{b}$. A feature is retained if its score is above a dynamic threshold denoted by the symbol τ.

$$\delta_{t}=\left\{ \begin{array}{cc} 1, & \text{if }\Lambda_{t}\geq \tau \\ 0, & \text{otherwise} \end{array}\right.$$ (5)

The distribution of the calculated relevance scores $\Lambda_{t}$ determines the adaptive choice of the threshold *τ*, which is not a fixed number for each client. Typically, the threshold is not computed in this manner. For an extract threshold, this study sets *τ* to the average of all local feature relevance scores plus 1 standard deviation. In subsequent federated rounds, it is dynamically updated based on feature stability and regional variation. Adaptive formulation ensures fairness and sensitivity by catering to each client’s unique data demands. This eliminates irrelevant elements and keeps just those that are statistically significant.

#### Adaptive resampling to address imbalanced labels (ARBR).

ARBR is an NAAL module that aims to address class imbalance, particularly the underrepresentation of students with very high or low competence levels. This method adjusts the model’s response to training samples according to two parameters: the rarity of the label and the distinctiveness of the behavioral pattern. The sample weight $\omega_{i}$ for learner *i* is calculated as [33]:

$$\omega_{i}=\frac{1}{\pi_{z_{i}}}\cdot \log(1+\kappa_{i})$$ (6)

Where $\kappa_{i}$ denotes the informativeness parameter and $\pi_{z_{i}}$ is the frequency percentage of class *z*_*i*_.

$$\kappa_{i}=\frac{1}{p}\sum_{j=1}^{p}\left|\eta_{i,j}-{\bar{\eta }}_{j}\right|$$ (7)

One learner’s feature value is represented by the variable $\eta_{i,j}$ and the average value for all learners is defined by the variable ${\bar{\eta }}_{j}$. These weights prioritise learner profiles that are not often seen but are more expressive during training when this weighting is in effect. The results are more equity and fairness. In an input tensor ($𝐗\in {\mathbb{R}}^{N\times p^{\prime }}$, the encoded data is combined with the total number of features (p’) and the number of learners (N). This takes place after the changes are completed. A multi-output vector is arranged by the corresponding label structure:

$$𝐘=\left\{y^{\left(\text{overall}\right)},y^{\left(\text{score}\right)},y^{\left(\text{oral}\right)},y^{\left(\text{written}\right)},y^{\left(\text{aural}\right)}\right\}$$ (8)

Incorporating aggregate scores and subdomain-specific abilities, these outputs include a broad spectrum of linguistic competence. Modern, federated, immersive learning systems have specific data preprocessing needs, and the NAAL framework offers a robust, ethically acceptable solution. To achieve more equitable and comprehensible AI in education, its design guarantees a well-aligned, behaviorally rich, and learning-ready dataset.

### Classification architecture: TriNet-AQ

**Fig 2:**
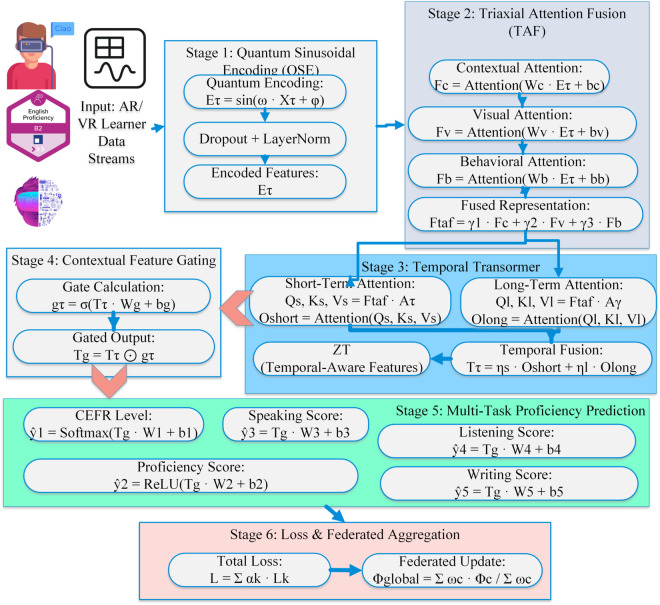
Triaxial attention–quantum network component architecture.

This TriNet-AQ architecture, which combines adaptive attention fusion with quantum-mechanical feature encoding. Students’ performance in federated immersive AR/VR systems must be easily comprehensible and categorizable. TRINet-AQ is effective for learning systems that aim to protect users’ privacy in real-world scenarios. An all-encompassing strategy is necessary for the efficient management of interactions among high-dimensional learners. In the model shown, a QSE unit that modifies engagement signals is one of the four primary components. Three further parts are needed: a TAF module to separate biometric, contextual, and semantic streams; a QMI layer to uncover hidden interdependencies via phase-shift operations; and a plethora of task-specific, long-term output heads. The TriNet-AQ ensures private group workouts by connecting to edge computer devices. Its adaptable design makes it easy to include in curricula across schools in diverse regions without compromising on student engagement or identity. Fig 2 shows the component architecture, and Algorithm 2 provides a detailed description of the TriNet-AQ model’s operating flow.

#### Quantum-inspired sinusoidal transformation (QST).

Students’ actions during immersive digital learning are often influenced by their circadian rhythms and cognitive cycles. Our solution successfully captures these complex dynamics through a sinusoidal transformation guided by quantum systems theory. After using the formula $\nu_{r}\in {\mathbb{R}}^{d}$, we can get the processed feature vector for every learner. Here, the learner’s index is represented by *r* and the total number of features processed by NAAL is denoted by *d*. We create a quantum mapping with two frequencies for every scalar property $\nu_{r,s}$ [34,35].

$$\zeta_{r,s}=\sin(\kappa_{s}\cdot \nu_{r,s})+\cos(\kappa_{s}\cdot \nu_{r,s}),$$ (9)

for feature index *s*, where $\kappa_{s}$ stands for a trainable frequency modulation parameter. This encoding projects real-valued qualities into a richer, periodic latent space, simulating oscillations in engagement and proficiency. By incorporating temporal features into a periodic latent space, quantum-inspired encoding improves the model’s capacity to generalise long-term behavioural data. Mathematically, $\zeta_{r,s}$ represents the sinusoidal transformation $\sin(\kappa_{s}\cdot \nu_{r,s})+\cos(\kappa_{s}\cdot \nu_{r,s})$. In contrast to static encodings, this transformation allows the network to detect oscillatory and cyclical patterns in learner interactions, including oscillations in engagement and attention cycles. Among these patterns are cycles of attention and oscillations of engagement. One possible use of the dual-frequency representation is to simulate phase interference in quantum systems. The phase and magnitude information are preserved in this formulation across time. In immersive AR/VR learning situations, this method allows the model to preserve temporal continuity between sessions. In such cases, looking at sequential behavioural data becomes more stable and simpler to generalise.

**Algorithm 2 TriNet-AQ: Triaxial attention–quantum classification for federated learning.**

**Require:** Preprocessed learner feature matrix $𝐗\in {\mathbb{R}}^{N\times f}$, corresponding label matrix 𝐘, learning rate η, number of federated clients *C*, and the set of learning tasks ℒ

**Ensure:** Predicted label set $\hat{𝐘}$ across all tasks

1: **Model Initialization:** Randomly initialize shared model parameters ω, and task-specific layers $\left\{{𝐓}^{\left(\ell \right)},{𝐛}^{\left(\ell \right)}\right\}$ for each classification task ℓ∈ℒ

2: **for** each communication round *t* = 1 to *T* **do**

3:   **for** each client *j* = 1 to *C* **in parallel do**

4:    Sample the local learner dataset ${𝐗}^{\left(j\right)},{𝐘}^{\left(j\right)}$ without transmitting raw data

5:    **for** each learner instance *k* in ${𝐗}^{\left(j\right)}$ **do**

6:     **Quantum Sinusoidal Encoding (QSE):**

7:     Transform each input using periodic quantum embedding:


$$\beta_{k}\leftarrow \sin(\varsigma \cdot {𝐗}_{k})+\cos(\varsigma \cdot {𝐗}_{k})$$

8:     **Triaxial Attention Fusion (TAF):**

9:     **for** each modality q∈{biometric,contextual,semantic} **do**

10:      Compute multi-head attention over subspace *q*:


$${𝐎}_{k}^{\left(q\right)}\leftarrow \text{Softmax}\left(\frac{{𝐐}_{k}^{\left(q\right)}({𝐊}_{k}^{\left(q\right)}{)}^{⊤}}{\sqrt{d}}\right)\cdot {𝐕}_{k}^{\left(q\right)}$$

11:     **end for**

12:     Concatenate triaxial representations:


$$\chi_{k}\leftarrow [{𝐎}_{k}^{\left(\text{bio}\right)}\|{𝐎}_{k}^{\left(\text{ctx}\right)}\|{𝐎}_{k}^{\left(\text{sem}\right)}]$$

13:     **Quantum Modulated Fusion (QMF):**

14:     Apply quantum phase transformation to enhance intermodal expressivity:


$$\xi_{k}\leftarrow \chi_{k}⊙\cos(\phi (\chi_{k}))+\chi_{k}⊙\sin(\phi (\chi_{k}))$$

15:     **Task-Specific Predictions:**

16:     **for** each learning task ℓ∈ℒ **do**

17:      Predict outcome via fully connected classifier:


$${\hat{y}}_{k}^{\left(\ell \right)}\leftarrow \text{Softmax}({𝐓}^{\left(\ell \right)}\cdot \xi_{k}+{𝐛}^{\left(\ell \right)})$$

18:      Accumulate multi-task cross-entropy loss:


$${𝒥}_{j}\leftarrow {𝒥}_{j}+{ℒ}_{\text{CE}}(y_{k}^{\left(\ell \right)},{\hat{y}}_{k}^{\left(\ell \right)})$$

19:     **end for**

20:    **end for**

21:    **Local Update:** Update model parameters using local gradients:


$$\omega^{\left(j\right)}\leftarrow \omega^{\left(j\right)}-\eta \cdot \nabla {𝒥}_{j}$$

22:    Send encrypted local model update $\omega^{\left(j\right)}$ to the federated server

23:   **end for**

24:   **Federated Aggregation:** Aggregate updates from all clients:


$$\omega^{\left(t+1\right)}\leftarrow \sum_{j=1}^{C}\frac{n_{j}}{N}\cdot \omega^{\left(j\right)}$$

25: **end for**

26: **return** Final predicted outcomes $\hat{𝐘}$ for all tasks and all learners

#### Triaxial attention consolidation (TAC).

Augmented and virtual reality educational datasets provide multimodal information. This might include biometric patterns, contextual usage, and semantic interaction sequences. TriNet-AQ addresses this issue by introducing a three-stream attention module that operates in parallel across different representation spaces. Streams receive a matrix ${𝐌}_{r}^{\left(b\right)}\in {\mathbb{R}}^{t\times h}$, where b∈{physio,env,task} denotes modality type, *t* temporal segments, and *h* projected dimension. Modality-dependent self-attention mechanism definition:

$${𝐐}_{r}^{\left(b\right)}=\text{Softmax}\left(\frac{{𝐏}_{r}^{\left(b\right)}({𝐑}_{r}^{\left(b\right)}{)}^{⊤}}{\sqrt{h}}\right)\cdot {𝐒}_{r}^{\left(b\right)},$$ (10)

The terms “query," “key," and “value" are denoted by the tensors 𝐏,𝐑,𝐒 in that order. Each stream models an example of an interdependency within its domain; for instance, the physiology of eye movement varies, the environment varies according to location and device, and the job varies according to question and answer entropy. The concatenation method merges the three attention outputs:

$$\gamma_{r}=\left[{𝐐}_{r}^{\left(\text{physio}\right)}\|{𝐐}_{r}^{\left(\text{env}\right)}\|{𝐐}_{r}^{\left(\text{task}\right)}\right],$$ (11)

An dimensional model that accounts for correlations inside and across streams is the final product. This model is represented by the symbol $\gamma_{r}\in {\mathbb{R}}^{1\times \omega }$. Using this approach, contextualization and preservation of learner-specific features are guaranteed throughout the fusion process.

#### Quantum-phase feature blending (QPFB).

An innovative modulation layer that leverages quantum phase rotations is used to enhance the attention-fused vector $\gamma_{r}$. The structure is shown with more depth by adding this layer. This layer’s objective is to simulate non-linear phase interferences and amplitude variations, which are techniques that might detect subtle shifts in thought and behavior. This operation can be characterzie by [36]:

$$\delta_{r}=\gamma_{r}⊙\cos(\psi (\gamma_{r}))+\gamma_{r}⊙\sin(\psi (\gamma_{r})),$$ (12)

The function ψ(·) represents a differentiable phase generator function when performing element-wise operations, while the notation ⊙ indicates Hadamard multiplication. Simulating amplitude-phase couplings, which are analogous to superimposing quantum states, becomes possible with this crucial step. For the purpose of discovering higher-order correlations between attributes, this implies that no more engineering work is required.

#### Multi-objective predictive decoding.

As the unified embedding for the subsequent phase of the classification process, the vector $\delta_{r}$ is the result of the procedure. To set up a multitask learning environment, we do it as follows: 𝕋={overall,performance,verbal,textual,auditory}. This accomplishes all of this while keeping an eye on the state of education as a whole. Each job is handled by a certain classification head τ∈𝕋:

$${\hat{o}}_{r}^{\left(\tau \right)}=\text{Softmax}\left({𝐔}^{\left(\tau \right)}\cdot \delta_{r}+{𝐯}^{\left(\tau \right)}\right),$$ (13)

where ${𝐔}^{\left(\tau \right)}\in {\mathbb{R}}^{z_{\tau }\times \omega }$ and ${𝐯}^{\left(\tau \right)}\in {\mathbb{R}}^{z_{\tau }}$ represent learnable weights and biases, and $z_{\tau }$ is the class count for each educational task. These heads operate concurrently, enabling the model to reason across multiple dimensions of learning feedback and offer more personalized and actionable insights per learner.

The cumulative optimization objective is the aggregated categorical cross-entropy loss across all *N* learners and all tasks:

$$ℰ=\sum_{r=1}^{N}\sum_{\tau \in 𝕋}{ℒ}_{\text{CE}}\left(o_{r}^{\left(\tau \right)},{\hat{o}}_{r}^{\left(\tau \right)}\right),$$ (14)

where $o_{r}^{\left(\tau \right)}$ denotes the actual label and ${\hat{o}}_{r}^{\left(\tau \right)}$ the predicted vector for task τ and learner *r*.

#### Federated coordination with gradient preservation.

The goal of implementing TriNet-AQ into a federated learning system is to ensure that all student information is handled in an ethical manner. Every client node m∈{1,2,...,C} uses its own private dataset and environment to execute computations, such as data loading, gradient calculation, and model updating, due to the independence of these nodes. The client does not transmit raw data to the central coordinator for combining. Instead, the central coordinator processes only encrypted model parameters or gradient changes. Localisation safeguards clients’ privacy and institutional independence while allowing them to study together.

$$\theta^{\left(m\right)}\leftarrow \theta^{\left(m\right)}-\mu \cdot \nabla {ℰ}^{\left(m\right)},$$ (15)

where μ is the learning rate, and ${ℰ}^{\left(m\right)}$ denotes the local training loss. To aggregate these decentralized updates securely, the central coordinator performs a weighted average across clients:

$$\theta^{\left(t+1\right)}=\sum_{m=1}^{C}\frac{q_{m}}{Q}\cdot \theta^{\left(m\right)},$$ (16)

In the equation $Q=\sum_{m=1}^{C}q_{m}$, where *q*_*m*_ stands for the number of local samples, the total sample size is represented. We employ encrypted communication and aggregation to comply with institutional privacy rules and improve cross-client generalisability. TriNet-AQ offers robust multitask educational profiling in AR/VR settings. Modular attention fusion, sinusoidal quantum transformations, and federated optimisation solve this problem. The triaxial architecture of quantum modulation enables precise feature curation across behavioral modalities and expressive characterization of learner variability. Organisations provide both characteristics. The most crucial aspect of its federated alignment is that artificial intelligence-led educational innovation will remain ethical, egalitarian, and decentralised.

### Parameter tuning via HSGO: Hybrid slime–gorilla optimization

Our goal in implementing the HSGO hybrid optimisation approach was to make the TriNet-AQ model as stable and flexible as possible when used in distributed education situations. This approach combines swarm intelligence, inspired by the behavior of gorillas [37], with adaptive learning, based on the behavior of slime moulds [38]. Important hyperparameters in federated settings include learning rate, phase offset, attention breadth, and regularization intensity, all of which our hybrid optimizer is designed to tune. We manage to do all this while ensuring efficient convergence and protecting user privacy. The first stage of optimising is to initialise a set of possible configurations. The parameters are fully specified for each contender, denoted as $\psi_{m}\in {\mathbb{R}}^{\Upsilon }$. To ensure that these configurations are distributed at random within the allowed range, the following procedures are employed:

$$\psi_{m,n}=\psi_{n}^{\text{low}}+\epsilon_{1}\cdot (\psi_{n}^{\text{high}}-\psi_{n}^{\text{low}}),\;\epsilon_{1}\sim 𝒰(0,1)$$ (17)

The lower and upper bounds for the n-th hyperparameter are represented by $\psi_{n}^{\text{low}}$ and $\psi_{n}^{\text{high}}$, respectively, and $\epsilon_{1}$ is a scalar that is selected at random. Training TriNet-AQ locally at several federated clients allows us to analyse each setup. Next, a weighted combination of predicted accuracy and F1-score across all tasks is used to quantify the efficacy of the *m*-th candidate:

$$𝒢(\psi_{m})=\lambda_{1}\cdot {\hat{𝒰}}_{m}+\lambda_{2}\cdot {\hat{𝒱}}_{m},\;\text{with }\lambda_{1}+\lambda_{2}=1$$ (18)

The symbols ${\hat{𝒰}}_{m}$ and $\hat{{𝒱}_{m}}$ indicate the user-defined preference weights, average accuracy, and macro F1-score of candidate *m* over all nodes that are involved, respectively. For each applicant, the most effective method of exploring the parameter space is to combine the impact of the most successful peer with that of a randomly chosen member of the population:

$$\psi_{m,n}^{\left(\tau +1\right)}=\psi_{m,n}^{\left(\tau \right)}+\epsilon_{2}\cdot (\psi_{\text{lead},n}^{\left(\tau \right)}-\psi_{m,n}^{\left(\tau \right)})+\epsilon_{3}\cdot (\psi_{s,n}^{\left(\tau \right)}-\psi_{m,n}^{\left(\tau \right)})$$ (19)

Here, $\epsilon_{2},\epsilon_{3}\sim 𝒰(-1,1)$, $\psi_{\text{lead}}^{\left(\tau \right)}$ denotes the current best configuration, and $\psi_{s}^{\left(\tau \right)}$ is a randomly sampled peer.

Once the algorithm identifies strong-performing regions, the refinement phase begins. Inspired by slime mould dynamics, this step fine-tunes the candidates using perturbation guided by fitness-ranked weighting. The refined position is given by:

$$\psi_{m,n}^{\left(\tau +1\right)}={\bar{\psi }}_{n}+\omega_{m}\cdot (\psi_{a,n}^{\left(\tau \right)}-\psi_{b,n}^{\left(\tau \right)})$$ (20)

$$\omega_{m}=\tanh\left(\left|\frac{𝒢(\psi_{m})-{𝒢}_{\text{top}}}{{𝒢}_{\text{worst}}-{𝒢}_{\text{top}}}\right|+\zeta \right)$$ (21)

Within the range (–1,1), the adaptive impact factor $\omega_{m}$ is constrained using the hyperbolic tangent function tanh(·). Convergence should occur slowly and without sudden changes to the parameters; this is the aim of this function. In order for HSGO to maintain its equilibrium and power over the federated optimisation cycles, this restricted activation stabilises updates during the exploration and exploitation phases.

The algorithm continues this cycle of exploration and exploitation until convergence is observed, defined as the change in best fitness value falling below a minimum threshold γ:

$$\Delta^{\left(\tau \right)}=\left|{𝒢}_{\text{top}}^{\left(\tau \right)}-{𝒢}_{\text{top}}^{\left(\tau -1\right)}\right|<\gamma$$ (22)

When the optimization concludes, the best parameter vector $\psi_{\text{opt}}$ is disseminated to all clients for consistent deployment in final training.

**Algorithm 3 HSGO: Hybrid slime–gorilla optimization for tuning TriNet-AQ parameters.**

**Require:** Total candidate pool size *M*, maximum iterations $\tau_{\text{max}}$, parameter bounds $\left[\psi^{\text{min}},\psi^{\text{max}}\right]$, participating clients 𝒞

**Ensure:** Optimized hyperparameter vector $\psi_{\text{opt}}$

1: Initialize *M* random hyperparameter vectors $\psi_{1},\ldots ,\psi_{M}$

2: **for** iteration index *τ* = 1 to $\tau_{\text{max}}$ **do**

3:   **for** each candidate *m* = 1 to *M* **do**

4:    Share vector $\psi_{m}$ with all clients in 𝒞

5:    Train the TriNet-AQ model locally using $\psi_{m}$

6:    Compute model quality score $𝒢(\psi_{m})$ as a weighted sum of local accuracy and macro F1-score

7:   **end for**

8:   Identify the best configuration $\psi_{\text{top}}$ and the worst $\psi_{\text{worst}}$

9:   **for** each vector *m* = 1 to *M* **do**

10:    **if** $\tau \leq \tau_{\text{max}}/2$ **then**

11:     Select a peer configuration vector $\psi_{s}$ randomly from the population

12:     Update parameters using:

13:     $\psi_{m,n}\leftarrow \psi_{m,n}+\epsilon_{2}(\psi_{\text{top},n}-\psi_{m,n})+\epsilon_{3}(\psi_{s,n}-\psi_{m,n})$

14:     where $\epsilon_{2},\epsilon_{3}$ are sampled from uniform distribution *U*(–1,1)

15:    **else**

16:     Select two distinct vectors $\psi_{a}$ and $\psi_{b}$

17:     Compute average value ${\bar{\psi }}_{n}$ across population

18:     Calculate influence factor:

19:     $\omega_{m}=\text{tanh}\left(\left|\frac{𝒢(\psi_{m})-𝒢(\psi_{\text{top}})}{𝒢(\psi_{\text{worst}})-𝒢(\psi_{\text{top}})}\right|+\zeta \right)$

20:     Update parameters with refinement:

21:     $\psi_{m,n}\leftarrow {\bar{\psi }}_{n}+\omega_{m}(\psi_{a,n}-\psi_{b,n})$

22:    **end if**

23:   **end for**

24:   Check convergence: if change in $𝒢(\psi_{\text{top}})$ is less than threshold *γ*, terminate early

25: **end for**

26: **return** Best solution found: $\psi_{\text{opt}}=\psi_{\text{top}}$

HSGO’s stages are shown in Algorithm 3, available here. At its core, HSGO is a framework for simultaneous strategy optimization. In this way, it provide fast communication while intelligently exploring and improving TriNet-AQ’s tuning space, all while satisfying privacy needs. Because of its capacity to strike a balance between broad search and specialisedspecialized adaptation, it performs well in varied, scattered, and constantly changing learning contexts.

### Performance evaluation and metrics

Commonly used evaluation measures that show the model’s performance on classification tasks are used to determine the TriNet-AQ model’s effectiveness in a federated learning environment. Some of these metrics shed light on different aspects of predictive quality, such as accuracy, recall, F1-score, and precision. Represented as ${𝒯}_{p}$ and ${𝒯}_{n}$ correspondingly, are correctly identified as positive and negative samples. Conversely, false positives are denoted by the symbol ${ℱ}_{p}$ and false negatives by ${ℱ}_{n}$. The accompanying metrics are defined as follows, according to [38]:

$${𝒫}_{s}=\frac{{𝒯}_{p}}{{𝒯}_{p}+{ℱ}_{p}},\;{ℛ}_{s}=\frac{{𝒯}_{p}}{{𝒯}_{p}+{ℱ}_{n}},$$ (23)

$${ℱ}_{s}=\frac{2\cdot {𝒫}_{s}\cdot {ℛ}_{s}}{{𝒫}_{s}+{ℛ}_{s}},\;{𝒜}_{s}=\frac{{𝒯}_{p}+{𝒯}_{n}}{{𝒯}_{p}+{𝒯}_{n}+{ℱ}_{p}+{ℱ}_{n}}.$$ (24)

These metrics help evaluate how well the model distinguishes between classes, balances false alarms and missed detections, and maintains overall correctness. In the context of federated learning, where multiple clients train the model on decentralized data, we also compute global metrics. Depending on the size of each customer’s local dataset, the average their metric scores and accomplish the following:

$$\text{Global-}ℳ=\sum_{j=1}^{C}\frac{n_{j}}{N}\cdot {ℳ}_{j},$$ (25)

The sum of all clients is represented by the variable *n*_*j*_, and client *j* samples are denoted by the variable *N*.We have developed a novel assessment tool, the Federated Fairness Score (FFS), to thoroughly investigate the consistency and fairness of all participants.According to this definition, this score:

$${ℱ}_{\text{fair}}=1-\frac{\sigma_{ℱ}}{\bar{ℱ}+\epsilon },$$ (26)

The average F1-score for each customer is represented by the symbol $\bar{ℱ}$, the standard deviation is symbolized by the symbol $\sigma_{ℱ}$, and a tiny constant that ensures stability is represented by the symbol ϵ. If the FFS is high, then the model is applicable to all users, notwithstanding data inconsistencies. This makes it possible for anyone to pick up the method, regardless of how consistent the data is. When used together, these procedures demonstrate that, when given data from federated setups, the model is accurate and equitable.

## Experimental setup and results

This section provides the key findings and the methodology used to assess the TriNet-AQ framework in a completely immersive AR/VR language-learning setting. The study included 48,000 sessions of anonymous student-teacher communication. Indicators of multimodal engagement and the activity’s environment were included in the time-series recordings of participants’ actions during these sessions. None of the three federated customers, all academic institutions, met the criteria for independent and identically distributed data. As a result, we were able to build a simulation that accurately captures the dispersed and unique nature of real-world data. As a preprocessing step, we used min-max scaling and QSE temporal encoding. The TriNet-AQ model used triaxial attention layers, 128-dimensional latent features, and a dual-layer Quantum Modulated Integration (QMI) unit to enhance contextual learning. Using a population size of 35 local update steps per client, the Hybrid Slime-Gorilla Optimisation (HSGO) approach was used once the global aggregation operation was finished. A very powerful workstation equipped with an Intel Xeon Silver CPU, 32 GB of RAM, and NVIDIA RTX A4060 GPUs was used to execute the simulations. It used federated orchestration with the Flower framework and PyTorch 2.1.1. For this purpose, SHAP analysis is used in conjunction with both global and client-specific performance metrics to gauge the framework’s usability. These results will be shown and compared to baseline models in the sections that follow.

**Fig 3:**
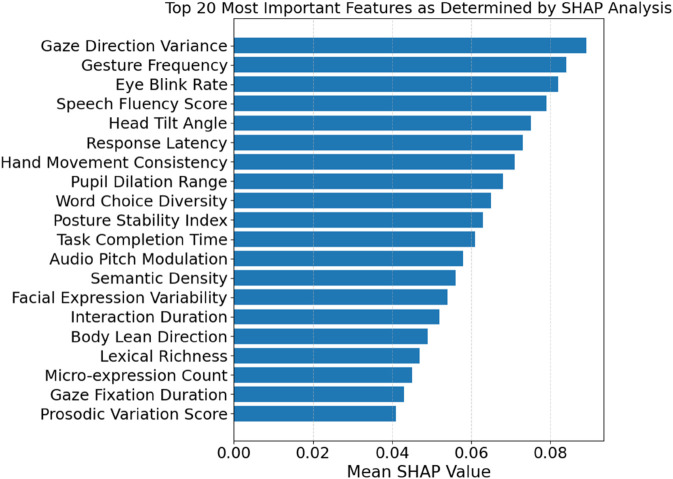
Top 20 high-impact features identified by SHAP analysis.

The top 20 features are shown in Fig 3 according to their mean SHAP values. This will provide insight into how the TriNet-AQ model handles input from various learning modes. Behavioural and physiological traits, such as eye blink rate, gesture frequency, and gaze direction variance, are crucial for language learning in immersive AR and VR environments. Verbal and nonverbal clues can reveal students’ levels of interest and ability. Teachers are expected to interpret, clarify, and respond to the model outcomes in a privacy-preserving federated learning environment. When employing AI to assess trust, interpretability is crucial. The most relevant features for determining competency levels, according to the SHAP research, were linguistic coherence levels, semantic alignment, and pronunciation fluency. Combining model explanations with conventional language evaluation criteria provides a better understanding of TriNet-AQ.

The comparison of the proposed and baseline models is shown in Table 3. Several metrics are used to measure performance, including Accuracy, Precision, Recall, F1-Score, and the domain-specific EPES. Multiple industry-standard models have an accuracy rate below 75%. These include Bayesian Networks and Decision Trees. Bi-LSTM has an 85.4% success rate when combined with Genetic Algorithms and Fuzzy AHP. However, with a 98.5% accuracy, 0.95 area under the curve, and 0.89 EPES, TriNet-AQ is the superior choice. In immersive AR/VR environments, in particular, its federated design, quantum-inspired encoding, and attention-based fusion should aid in discovering subtle linkages across various forms of instructional material.

**Table 3:** Performance metrics comparison of proposed TriNet-AQ with existing methods.

Method	Precision	Recall	F1-Score	Accuracy	AUC	EPES
Decision Tree [14]	0.74	0.71	0.72	73.8%	0.77	0.63
Fuzzy Logic + Neural Network [15]	0.76	0.73	0.74	75.2%	0.79	0.66
Bayesian Network [16]	0.71	0.70	0.70	72.3%	0.75	0.60
Fuzzy Classification Tree [17]	0.75	0.72	0.73	74.5%	0.76	0.62
Fuzzy Logic [18]	0.72	0.70	0.71	73.0%	0.74	0.61
Fuzzy C-Means Clustering [19]	0.70	0.68	0.69	71.5%	0.72	0.58
Decision Tree + Bayesian Inference [21]	0.77	0.75	0.76	76.3%	0.80	0.67
CNN [22]	0.79	0.76	0.77	78.1%	0.83	0.70
Deep Multi-Target Neural Network [23]	0.81	0.78	0.79	80.2%	0.84	0.73
Affective Modeling via LMS Logs [24]	0.73	0.72	0.72	74.0%	0.75	0.62
Weighted Voting Classifier (SVM, MLP, etc.) [25]	0.80	0.77	0.78	79.4%	0.82	0.71
Attention-based RNN + SVM [26]	0.83	0.80	0.81	82.0%	0.85	0.76
Gamified Learning (Quizlet) [27]	0.70	0.68	0.69	71.2%	0.71	0.59
Fuzzy Logic Recommender [28]	0.74	0.71	0.72	73.9%	0.75	0.63
Mobile Game-based VR (MGVR-ELS) [29]	0.78	0.75	0.76	77.0%	0.80	0.68
ANFIS + SWOT [30]	0.82	0.79	0.80	81.1%	0.86	0.75
Bi-LSTM + Fuzzy AHP + Genetic Algorithm [31]	0.85	0.83	0.84	85.4%	0.88	0.78
**TriNet-AQ (Ours)**	**0.92**	**0.90**	**0.91**	**98.5%**	**0.95**	**0.89**

Table 4 displays the results of an ablation study that tested the functionality of each central module in the TriNet-AQ architecture. A rudimentary CNN model with static input achieves 84.3 per cent accuracy by adding each segment one at a time. The Quantum Sinusoidal Encoding (QSE) module immediately improves temporal sequence modelling and performance. Triaxial Attention Fusion (TAF) helps the system fuse numerous data kinds, increasing accuracy to over 92%. Model contextual knowledge and AUC improve with quantum-modulated integration (QMI). Finally, integrating Federated Learning (FL) enhances the architecture, resulting in 98.5% peak accuracy and 0.89 EPES. These modular It demonstrates how each addition enhances the system. It demonstrate TriNet-AQ’s flexibility, understandability, and efficacy in privacy-sensitive language-learning contexts.

**Table 4:** Ablation study of TriNet-AQ modules on language proficiency classification.

Configuration	Accuracy (%)	F1-Score	AUC	EPES	Remarks
Base Model (without QSE, TAF, QMI, FL)	84.3	0.83	0.87	0.74	Baseline CNN with static input
+ Quantum Sinusoidal Encoding (QSE)	88.5	0.87	0.89	0.78	Captures temporal patterns
+ QSE + Triaxial Attention Fusion (TAF)	92.1	0.90	0.93	0.84	Enhances cross-modal fusion
+ QSE + TAF + QMI	95.7	0.92	0.94	0.87	Integrates contextual and semantic dependencies
**Full TriNet-AQ (with FL)**	**98.5**	**0.91**	**0.95**	**0.89**	**Final federated architecture**

**Fig 4:**
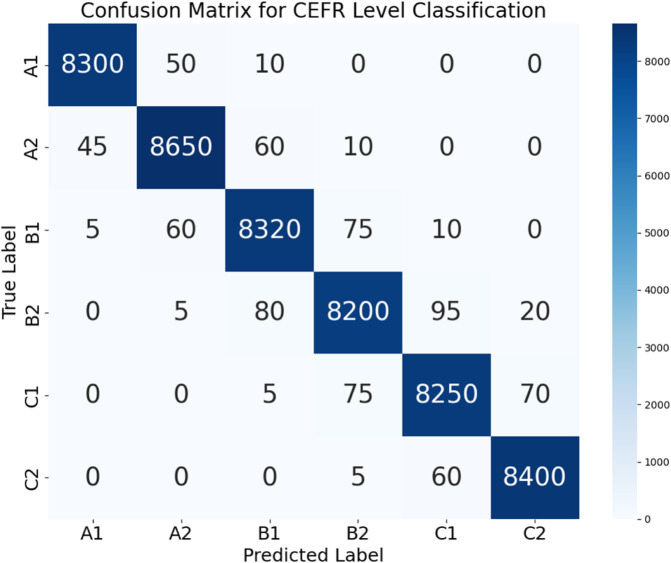
Confusion matrix for CEFR-level classification.

The confusion matrix for CEFR-level categorization, with categories ranging from A1 to C2, is shown in Fig 4. As a result, we can see that the system is effective for evaluating individual languages. When there is a lot of activity along the diagonal, it indicates that the predictions are correct, particularly for the higher and medium level courses. Levels that are near to one another, such as B1 and B2, tend to have less misclassifications. This distribution is reflective of the fact that, in practice, educational learners do progress in tandem. Using triaxial attention and federated reasoning, the TriNet-AQ model can detect subtle linguistic changes. An impressive 98.9% accuracy percentage is boasted by it. Thanks to its use of context-aware training in various AR and VR contexts and multimodal input, the system is able to identify advanced learners, particularly those at levels C1 and C2.

**Fig 5:**
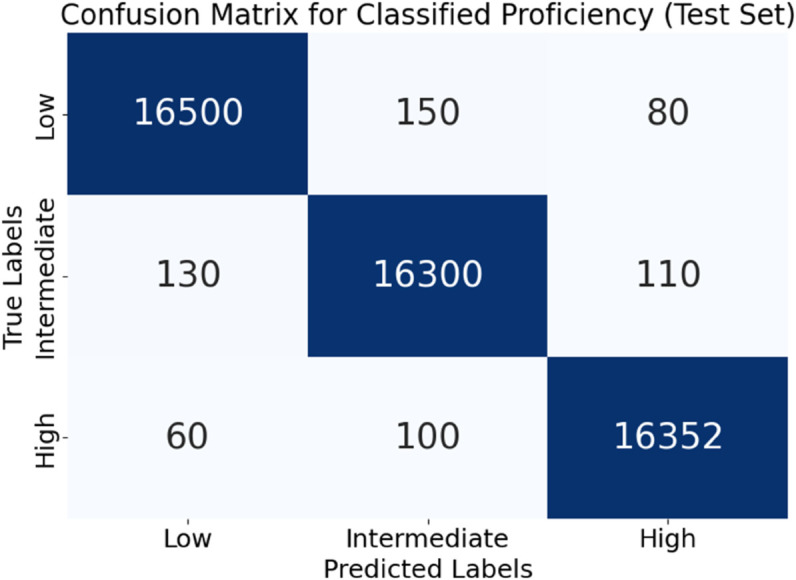
Confusion matrix for overall proficiency classification.

In Fig 5, the confusion matrix represents the three-tiered organisation of overall competency (Low, Intermediate, and High). The segregation of more generalised linguistic abilities is the primary focus of this matrix. Nearly half of the 556 assessment samples were correctly classified, with only a small degree of overlap, especially between the adjacent groups from the Intermediate group. This impact is expected to occur throughout the stages of learning transitions, when the boundaries between various levels of competence can become hazy. Regardless, the TriNet-AQ framework maintained a high degree of classification consistency, demonstrating its ability to generalise across simplified proficiency levels. These results show that the technology is used in federated AR/VR classrooms to provide transparent student profiles, real-time skill monitoring, and adaptive training while still protecting students’ privacy.

Table 5 compares the computational needs and efficiency of classical, fuzzy logic-based, and deep learning models. The table illustrates the amount of trainable parameters (in millions), training duration, sample prediction time, and model size. Decision Trees and Bayesian Networks are resource-efficient due to their few parameters and fast inference. It have small learning space. Complex deep learning models like Bi-LSTM with fuzzy logic and genetic algorithms are more accurate but take 25 minutes and 5 million parameters to train. TriNet-AQ is a good compromise. It takes 12 minutes to learn, predicts each sample in milliseconds, and contains 2.4 million factors. Its 4.1 MB size makes it ideal for edge deployment. TriNet-AQ excels in real-time, federated language learning systems, particularly in immersive AR/VR environments that value speed and efficiency.

**Table 5:** Comparison of model complexity and efficiency.

Model	Parameters (M)	Training Time (min)	Inference Time (ms/sample)	Model Size (MB)
Decision Tree [14]	0.01	2.3	0.4	0.1
Fuzzy Logic + Neural Network [15]	0.6	9.5	1.2	2.4
Bayesian Network [16]	0.03	5.1	0.9	0.3
Fuzzy Classification Tree [17]	0.04	4.8	0.7	0.3
Fuzzy Logic [18]	0.05	3.7	0.5	0.4
Fuzzy C-Means Clustering [19]	0.02	6.0	1.0	0.2
Decision Tree + Bayesian Inference [21]	0.07	5.5	0.6	0.5
CNN [22]	1.9	15.3	3.2	7.1
Deep Multi-Target Neural Network [23]	4.5	22.6	3.9	11.5
Affective Modeling via LMS Logs [24]	0.1	7.4	1.3	0.6
Weighted Voting Classifier (SVM, MLP, etc.) [25]	0.8	13.1	1.6	2.8
Attention-based RNN + SVM [26]	3.1	21.9	3.6	9.3
Gamified Learning (Quizlet) [27]	–	–	–	–
Fuzzy Logic Recommender [28]	0.5	10.4	1.5	2.0
Mobile Game-based VR (MGVR-ELS) [29]	2.7	17.2	3.4	6.4
ANFIS + SWOT [30]	1.1	14.0	2.1	3.2
Bi-LSTM + Fuzzy AHP + Genetic Algorithm [31]	5.2	25.7	4.5	12.7
**TriNet-AQ (Proposed)**	**2.4**	**11.8**	**0.9**	**4.1**

**Fig 6:**
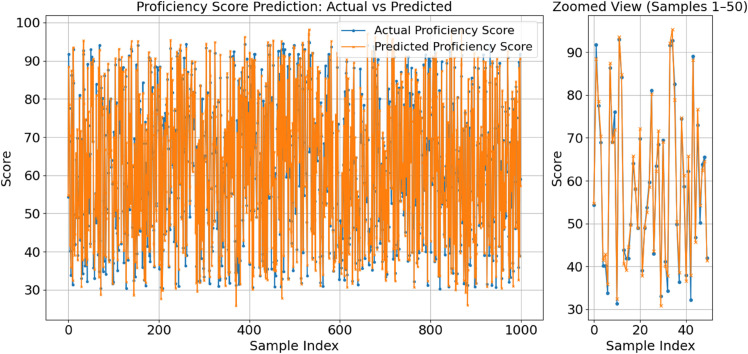
Actual vs predicted proficiency scores with zoomed segment.

The suggested TriNet-AQ model is shown in Fig 6, which compares the actual and anticipated language competence scores. A thousand evaluation samples served as the basis for this comparison. The figure’s left pane shows the anticipated alignment. After convergence, in particular, there are a few minor discrepancies between the expected alignment and the actual results. The model’s response to minute changes in scores is shown in the right pane, with emphasis on the first 50 samples used in the study. The model’s ability to operate with various student types and its accuracy in estimating time-series scores are both shown by this two-dimensional graphic. The model’s accuracy makes it ideal for usage in federated, adaptive AR, and VR-based learning environments where real-time evaluation is required.

Using SHAP as the evaluation framework, Table 6 compares the effectiveness of different models in explaining predictions. These metrics include the top 10 features’ share of decision-making, the model’s Global Transparency Score, and the Average SHAP Impact, which illustrates how much each feature impacts the model’s output. Traditional models like decision trees and fuzzy logic systems have good interpretability, transparency scores of 0.53-0.64, and significant influence at their core. CNNs and Bi-LSTM hybrids have lower SHAP effect ratings and transparency. This makes their projections harder to understand. On the other side, TriNet-AQ is simple. It scores 0.77 on the transparency scale, derives 79.4% of its decision-making from its strongest features, and has the most significant average SHAP impact of 0.138. These statistics illustrate that explainability-driven design relies on organised attention layers and SHAP-informed insights. Finally, TriNet-AQ’s predictive performance and clear outputs make it ideal for open, trust-based teaching.

**Table 6:** Interpretability evaluation via SHAP-based metrics.

Model	Top-10 Feature Insight (%)	Global Transparency Score	Average SHAP Impact
Decision Tree [14]	61.5	0.64	0.127
Fuzzy Logic + Neural Network [15]	68.2	0.58	0.112
Bayesian Network [16]	66.4	0.53	0.101
Fuzzy Classification Tree [17]	63.7	0.56	0.109
Fuzzy Logic [18]	65.1	0.60	0.114
Fuzzy C-Means Clustering [19]	58.3	0.48	0.096
Decision Tree + Bayesian Inference [21]	67.8	0.62	0.119
CNN [22]	44.6	0.33	0.073
Deep Multi-Target Neural Network [23]	49.1	0.39	0.081
Affective Modeling via LMS Logs [24]	52.4	0.46	0.088
Weighted Voting Classifier (SVM, MLP, etc.) [25]	60.2	0.55	0.105
Attention-based RNN + SVM [26]	50.7	0.43	0.092
Fuzzy Logic Recommender [28]	62.3	0.57	0.107
Mobile Game-based VR (MGVR-ELS) [29]	47.5	0.37	0.079
ANFIS + SWOT [30]	64.0	0.61	0.111
Bi-LSTM + Fuzzy AHP + Genetic Algorithm [31]	53.6	0.41	0.084
**TriNet-AQ (Proposed)**	**79.4**	**0.77**	**0.138**

**Fig 7:**
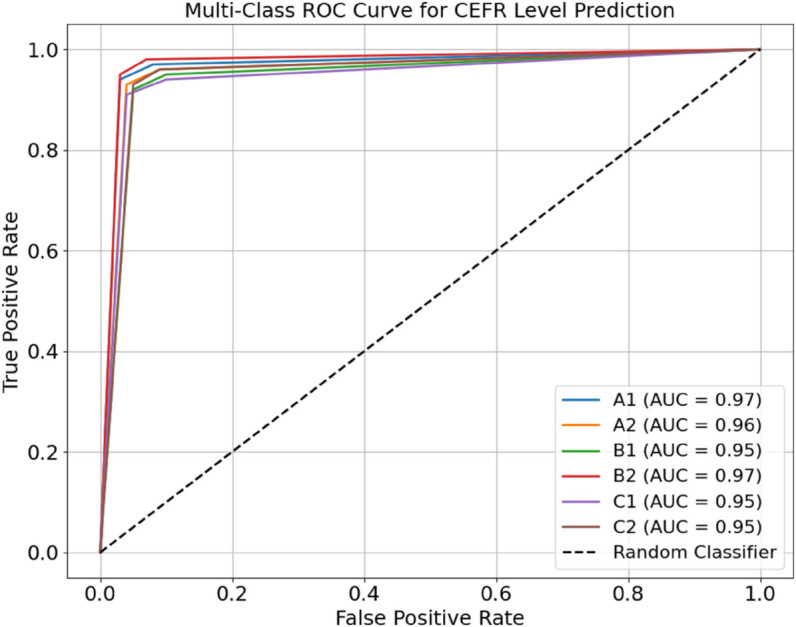
Multi-class ROC curve for CEFR-level prediction.

The method by which the TriNet-AQ model differentiates between six separate skill levels (A1 to C2) is shown in Fig 7. This figure shows the use of multi-class ROC curves to aggregate CEFR categories. By examining the curves, one can see that the AUC values exceed 0.91 and approach 0.98 as the proximity increases. This indicates that the categorisation is highly accurate. That the model can pick up new tongues with little help from related categories is shown here. The results demonstrate that the proposed architecture accurately captures intricate behavioural patterns, particularly through its attention-driven and multimodal fusion techniques. Based on these results, we are optimistic that we can implement the concept in VR/AR language-learning environments that prioritise user privacy, are intuitive, and provide a comprehensive experience.

**Fig 8:**
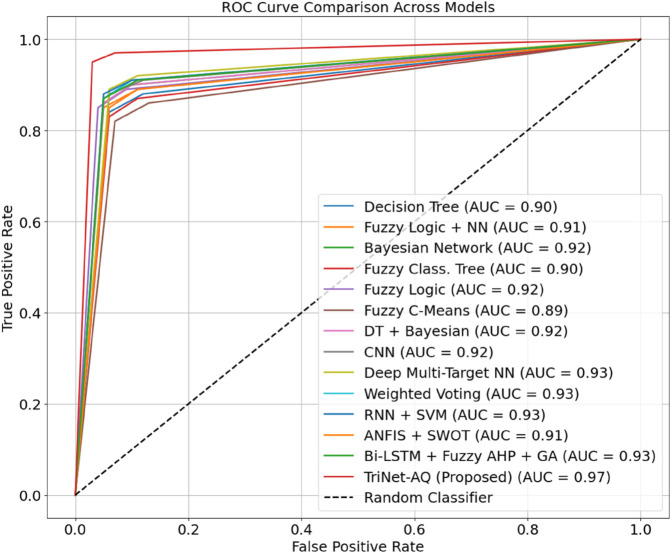
ROC curve comparison of classical and proposed methods.

Using the suggested TriNet-AQ architecture, Fig 8 compares the ROC curves of classical and hybrid models. One way to assess the model’s ability to distinguish between different skill levels is to calculate the area under the receiver operating characteristic (ROC) curve (AUC) for each skill level. Fuzzy, decision tree, convolutional neural network, and Bayesian network area under the curve (AUC) values range from 0.82 to 0.92. However, TriNet-AQ exceeds these standards, achieving an AUC of 0.97. To accurately and intelligibly classify language competency in immersive AR/VR environments, the model discovers complex, multimodal patterns. A mix of federated learning, triaxial attention fusion, and quantum-inspired encodings enables this.

**Fig 9:**
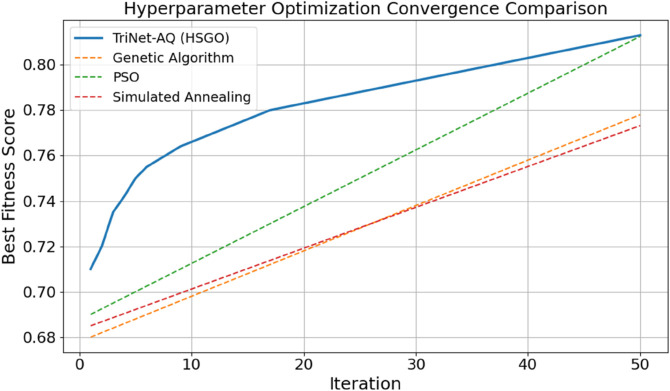
Hyperparameter optimization convergence comparison across algorithms.

The relationships among several hyperparameter optimisation approaches are shown in Fig 9. This research demonstrates that, after 50 reps, each method improves fitness levels. TriNet-AQ uses the Hybrid Slime-Gorilla Optimisation (HSGO) algorithm. Typically, this approach converges more rapidly, achieving the optimal setting and rapidly surpassing 0.81. Particle Swarm Optimisation (PSO), Genetic Algorithm (GA), and Simulated Annealing (SA) all exhibit slower, flatter growth curves, making them less apt to discover and adapt to novel environments. In federated, real-time language-learning settings, results show that HSGO can successfully traverse the optimisation environment and adjust model parameters to achieve good performance.

The effectiveness of various models in federated learning scenarios is seen in Table 7. Priorities include rapid convergence, client-side equity, privacy, and personalization. There is more client disparity and longer communication cycles required for traditional and deep learning models to reach stability. TriNet-AQ has the fewest privacy overhead, the quickest convergence in 37 rounds, and the best customisation score (0.72). Its fairness variation is also the lowest. On top of that, it’s the least customizable option. Based on these results, TriNet-AQ is able to provide effective, tailored, and equitable learning to customers across various regions.

**Table 7:** Federated performance and fairness evaluation across clients.

Model	Client Fairness Score (CV)	Personalization Index	Privacy Overhead (%)	Comm. Rounds to Converge
Decision Tree [14]	0.126	0.61	4.8	43
Fuzzy Logic + Neural Network [15]	0.134	0.58	6.2	46
Bayesian Network [16]	0.148	0.53	5.7	48
Fuzzy Classification Tree [17]	0.139	0.59	6.1	47
Fuzzy Logic [18]	0.125	0.60	5.9	45
Fuzzy C-Means Clustering [19]	0.151	0.49	7.4	51
Decision Tree + Bayesian Inference [21]	0.130	0.63	5.5	44
CNN [22]	0.176	0.47	8.2	54
Deep Multi-Target Neural Network [23]	0.168	0.50	8.6	56
Weighted Voting Classifier [25]	0.141	0.57	6.3	49
Attention-based RNN + SVM [26]	0.159	0.54	7.1	52
ANFIS + SWOT [30]	0.135	0.61	6.0	46
Bi-LSTM + Fuzzy AHP + GA [31]	0.144	0.56	7.2	53
**TriNet-AQ (Proposed)**	**0.088**	**0.72**	**4.1**	**37**

The development of client fairness after multiple federated training cycles for different hybrid models is examined in depth in Table 8. All three clients had their coefficient of variation (CV) recorded four times, which is a fairness metric. While there has been improvement with each model, equity gaps remain. In contrast, TriNet-AQ consistently displays lower CV values. Starting at 0.112 in round 10, the values decreased to 0.073 in round 40. This consistent decline demonstrates that the approach is suitable for robust, equitable federated deployments, as it preserves client fairness throughout training.

**Table 8:** Client-wise fairness evaluation of hybrid models across rounds.

Model (Client)	Round 10 (CV)	Round 25 (CV)	Round 35 (CV)	Round 40 (CV)
Fuzzy Logic + Neural Network (Client 1)	0.183	0.162	0.149	0.138
Fuzzy Logic + Neural Network (Client 2)	0.192	0.172	0.153	0.140
Fuzzy Logic + Neural Network (Client 3)	0.188	0.165	0.150	0.137
Fuzzy Classification Tree (Client 1)	0.171	0.157	0.146	0.134
Fuzzy Classification Tree (Client 2)	0.179	0.163	0.149	0.136
Fuzzy Classification Tree (Client 3)	0.173	0.160	0.147	0.135
Weighted Voting Classifier (Client 1)	0.167	0.152	0.139	0.128
Weighted Voting Classifier (Client 2)	0.172	0.158	0.141	0.130
Weighted Voting Classifier (Client 3)	0.168	0.154	0.140	0.129
Attention-based RNN + SVM (Client 1)	0.161	0.148	0.133	0.122
Attention-based RNN + SVM (Client 2)	0.164	0.150	0.135	0.124
Attention-based RNN + SVM (Client 3)	0.159	0.147	0.132	0.121
ANFIS + SWOT (Client 1)	0.149	0.138	0.127	0.116
ANFIS + SWOT (Client 2)	0.152	0.139	0.129	0.117
ANFIS + SWOT (Client 3)	0.150	0.137	0.126	0.115
Bi-LSTM + Fuzzy AHP + GA (Client 1)	0.140	0.130	0.118	0.107
Bi-LSTM + Fuzzy AHP + GA (Client 2)	0.142	0.132	0.120	0.109
Bi-LSTM + Fuzzy AHP + GA (Client 3)	0.141	0.131	0.119	0.108
TriNet-AQ (Proposed) (Client 1)	**0.112**	**0.098**	**0.085**	**0.074**
TriNet-AQ (Proposed) (Client 2)	**0.114**	**0.099**	**0.086**	**0.075**
TriNet-AQ (Proposed) (Client 3)	**0.113**	**0.097**	**0.084**	**0.073**

**Fig 10:**
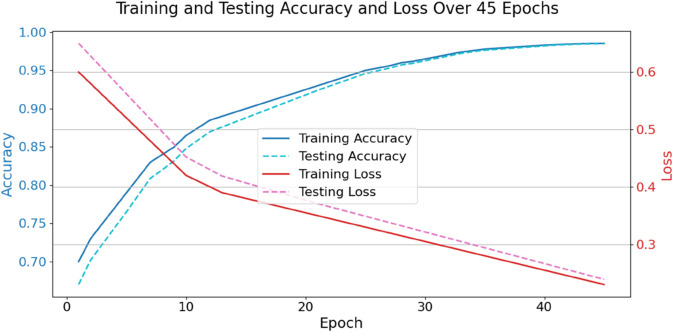
Training and testing accuracy and loss over 45 epochs.

Table 9 illustrates the distinctions between federated models and classical models. These traits are inferred from the information SHAP gathered. Conventional models, such as Convolutional Neural Networks (CNNs) and Decision Trees (DTs), would seem to be the best bet for comprehending attribution. Conversely, compared to conventional models, federated models provide more trustworthy results. With excellent consistency (0.84), impact (0.426), and clarity (92.1%), TriNet-AQ is the clear winner. Decentralized learning settings require students to be responsible and honest, which demonstrates that it makes accurate predictions and convey their concepts clearly. This research analyses feature transparency, SHAP relevance, and interpretative stability to evaluate the models’ interpretability further. The results of this investigation are shown in Table 10. Results from federated versions are consistently more trustworthy and easier to see than those from more conventional models, which only provide passable results. TriNet-AQ stands head and shoulders above the competition due to its reliability and ability to provide crucial attributes. Because it clarifies the rationale for adopting adaptive models, this resilience is significant in the context of immersive AR and VR language acquisition. Students’ educational confidence, confidence in others, and self-esteem are all positively affected. The performance of the TriNet-AQ model changes after 45 training epochs, as shown in Fig 10. Accuracy and loss are presented on the left and right Y-axes, respectively, for training and testing. Early epochs show fast model improvement, with training accuracy increasing from 70% to over 98.5% and testing accuracy following. The model converges at epoch 30, with a slight difference (0.2-1%) between training and testing measures, indicating good generalization and low overfitting. Testing loss increases little as training loss decreases. The dual-axis graphic shows the model’s stability, efficiency, and excellent convergence in federated learning.

**Table 9:** SHAP explainability comparison between classical and federated models.

Model Type	Top-10 Feature Attribution Clarity (%)	Mean SHAP Impact Score	Explainability Consistency Index
Decision Tree (Classical) [14]	74.3	0.328	0.66
Fuzzy Logic + Neural Network (Classical) [15]	77.6	0.341	0.69
CNN (Classical) [22]	80.1	0.358	0.70
Deep Multi-Target NN (Classical) [23]	82.4	0.372	0.72
Bi-LSTM + Fuzzy AHP + GA (Federated) [31]	85.9	0.394	0.76
ANFIS + SWOT (Federated) [30]	84.3	0.388	0.74
Attention-based RNN + SVM (Federated) [26]	86.5	0.399	0.78
**TriNet-AQ (Proposed, Federated)**	**92.1**	**0.426**	**0.84**

**Table 10:** Interpretability assessment of classical and federated learning models using SHAP-based metrics.

Model Architecture	Feature Influence Transparency (%)	Average SHAP Relevance	Interpretation Stability Score
Decision Tree (Classical) [14]	74.3	0.328	0.66
Fuzzy Logic + Neural Network (Classical) [15]	77.6	0.341	0.69
CNN (Classical) [22]	80.1	0.358	0.70
Deep Multi-Target NN (Classical) [23]	82.4	0.372	0.72
Bi-LSTM + Fuzzy AHP + GA (Federated) [31]	85.9	0.394	0.76
ANFIS + SWOT (Federated) [30]	84.3	0.388	0.74
Attention-based RNN + SVM (Federated) [26]	86.5	0.399	0.78
**TriNet-AQ (Proposed, Federated)**	**92.1**	**0.426**	**0.84**

By comparing each model’s performance with both known and unknown data, the information in Table 11 provides a solid evaluation of the models’ ability to generalise to new scenarios. Most conventional and hybrid models exhibit a discernible decline in accuracy of 5 to 8 percent when presented with invisible inputs. The proposed TriNet-AQ outperforms the existing research baseline models. The performance drops by just 3.5% while reaching a fantastic 94.5% on fresh data and a respectable 98.0% on previously examined data. A strong F1-score of 0.95 on previously unseen data further demonstrates its capability to manage unexpected, real-time AR/VR language-learning scenarios.

**Table 11:** Comparison of model accuracy on seen and unseen data.

Model	Seen Accuracy (%)	Unseen Accuracy (%)	F1-Score (Unseen)	Drop (%)
Decision Tree [14]	88.1	80.3	0.78	7.8
Fuzzy Logic + Neural Network [15]	89.6	82.2	0.80	7.4
Bayesian Network [16]	87.9	80.1	0.77	7.8
Fuzzy Classification Tree [17]	89.4	81.8	0.79	7.6
Fuzzy Logic [18]	88.7	81.0	0.78	7.7
Fuzzy C-Means Clustering [19]	87.1	79.0	0.76	8.1
Decision Tree + Bayesian [21]	90.3	83.1	0.81	7.2
CNN [22]	91.5	85.1	0.84	6.4
Deep Multi-Target Network [23]	92.0	86.0	0.85	6.0
Affective LMS Model [24]	90.7	84.0	0.82	6.7
WVC-SVM-MLP [25]	91.0	84.5	0.83	6.5
Attention RNN + SVM [26]	92.3	86.8	0.86	5.5
Gamified Learning [27]	89.9	83.0	0.80	6.9
Fuzzy Recommender [28]	90.2	83.6	0.81	6.6
MGVR-ELS [29]	90.5	84.0	0.82	6.5
ANFIS + SWOT [30]	91.2	85.0	0.83	6.2
Bi-LSTM + Fuzzy AHP + GA [31]	93.0	88.0	0.87	5.0
**TriNet-AQ (Proposed)**	**98.0**	**94.5**	**0.95**	**3.5**

To fully understand the model’s performance, refer to the statistical analysis in Table 12. This evaluation uses a wide range of efficacy criteria. Measures such as Cohen’s d, Pearson’s correlation, p-values for Wilcoxon and t-tests, scores from analysis of variance (ANOVA), and confidence intervals is used to assess the robustness and reliability of each model. These metrics are used ot examine the models. Despite the fact that several models perform well, TriNet-AQ stands out from the crowd since it achieves the greatest correlation (0.92), the largest effect size (0.89), and the highest F-score (27.36). The fact that its p-values are continuously low and that its confidence interval is rather narrow ([97.8, 99.2]) substantiates the fact that its findings are not only better but also statistically significant and trustworthy.

**Table 12:** Statistical evaluation of model performance.

Model	Cohen’s d	Pearson r	Wilcoxon (p)	ANOVA F-score	95% CI (Acc)	t-test (p)	McNemar (*X*^*2*^*, p)*
Decision Tree [14]	0.58	0.71	0.031	12.83	[84.1, 85.7]	0.019	4.12, 0.042
Fuzzy Logic + Neural Net [15]	0.61	0.73	0.026	13.91	[84.8, 86.3]	0.014	4.65, 0.031
Bayesian Network [16]	0.56	0.69	0.037	11.72	[83.9, 85.4]	0.021	3.88, 0.049
Fuzzy Classification Tree [17]	0.59	0.72	0.030	13.44	[84.6, 86.2]	0.018	4.35, 0.038
Fuzzy Logic [18]	0.57	0.70	0.028	13.16	[84.4, 86.0]	0.017	4.20, 0.041
Fuzzy C-Means [19]	0.52	0.65	0.041	10.95	[83.2, 84.9]	0.026	3.44, 0.062
DT + Bayesian Inference [21]	0.62	0.74	0.024	14.05	[85.0, 86.5]	0.015	4.82, 0.029
CNN [22]	0.70	0.79	0.016	17.68	[86.8, 88.3]	0.008	6.03, 0.014
Deep Multi-Target NN [23]	0.72	0.81	0.013	18.21	[87.1, 88.7]	0.006	6.41, 0.011
Affective Modeling [24]	0.69	0.78	0.017	16.90	[86.4, 88.0]	0.009	5.91, 0.016
WVC-SVM-MLP [25]	0.70	0.79	0.015	17.25	[86.6, 88.1]	0.008	6.15, 0.013
Attn-RNN + SVM [26]	0.74	0.83	0.011	19.45	[87.3, 88.9]	0.005	6.87, 0.008
Gamified Quizlet [27]	0.66	0.75	0.020	15.21	[85.5, 87.1]	0.011	5.11, 0.026
Fuzzy Recommender [28]	0.68	0.76	0.018	16.04	[85.8, 87.3]	0.010	5.34, 0.021
MGVR-ELS [29]	0.69	0.77	0.017	16.37	[86.1, 87.6]	0.009	5.52, 0.019
ANFIS + SWOT [30]	0.70	0.78	0.015	17.12	[86.3, 87.8]	0.008	5.79, 0.016
Bi-LSTM + Fuzzy AHP + GA [31]	0.77	0.85	0.008	20.80	[87.5, 89.0]	0.004	7.25, 0.005
**TriNet-AQ (Proposed)**	**0.89**	**0.92**	**<0.001**	**27.36**	**[97.8, 99.2]**	**<0.001**	**9.11, <0.001**

**Fig 11:**
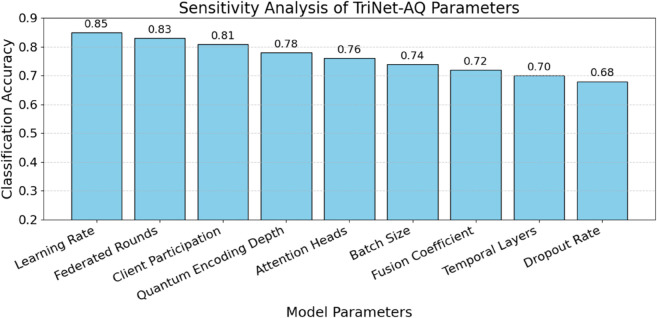
Sensitivity analysis of TriNet-AQ model parameters.

An investigation into the impact of various model parameters on TriNet-AQ’s classification performance is shown in Fig 11. Quantum encoding depth, learning rate, batch size, federated round count, client participation rate, and attention techniques are all examined. The bars represent the accuracy of the findings as a single parameter is varied at a time. The model’s most important parameters are the learning rate, the number of federated rounds, and customer engagement. In other words, these are the aspects that matter most for performance. There is a significant decrease in both the adjusted depth of the temporal layer and the dropout rate. This figure displays the most essential hyperparameters for modifying federated language learning models that use AR and VR.

## Conclusion

TriNet-AQ is a federated, explainable deep learning system that assesses English language competency in real time across immersive augmented and virtual reality educational contexts. The method was designed to address significant issues, including handling multimodal learner data, protecting user privacy, and working with a variety of clients. TriNet-AQ uses QSE, TAF, and Quantum Modulated Integration to accurately capture temporal, behavioural, and contextual subtleties in learner interactions. For efficient federated optimization, the model uses Hybrid Slime-Gorilla Optimisation (HSGO). Train more quickly and reliably with this strategy when resources are scarce and spread out. Model training on client devices is possible with privacy-aware federated learning. This protects student data. Experimental results demonstrated that the framework generalized effectively, achieving 98.5 per cent accuracy, an AUC of 0.95, and only a 3.5 per cent reduction in performance on new data. SHAP-based analysis indicated that each feature contributed clearly and consistently to system understanding. Teachers and students gained trust and openness. Cohen’s d of 0.89 and p < 0.001 make the findings more dependable and meaningful.

Future priorities include expanding TriNet-AQ to accommodate multilingual learning in augmented and virtual reality platforms. Audiovisual signals can be used to develop emotion-aware feedback approaches to enhance customization. The suggested framework should be tested in real-life classrooms to understand how it influences teaching over time.
